# Relaxant effect of BAY 41-2272 and BAY 58-2667 in the gastrointestinal tract of apo-sGC mice

**DOI:** 10.1186/1471-2210-11-S1-P19

**Published:** 2011-08-01

**Authors:** Sarah Cosyns, Johannes-Peter Stasch, Peter Brouckaert, Romain A Lefebvre

**Affiliations:** 1 Heymans Institute of Pharmacology, Ghent University, Ghent, Belgium; 2 Cardiovascular Research, Bayer HealthCare, Wuppertal, Germany; 3 Department of Molecular Biomedical Research, VIB, Ghent, Belgium

## Background

In vascular tissue, relaxation by the NO-independent but heme-dependent sGC stimulator BAY 41-2272 is reduced when sGC is oxidized and finally loses its heme group, while that by the NO- and heme-independent sGC activator BAY 58-2667 (cinaciguat) is increased. Whether this also applies in the gastrointestinal tract has not been investigated. In apo-sGC mice, his at position 105 of sGCβ1 is mutated to phe; both sGC isoforms (α1β1 and α2β1) are heme-deficient and can no longer be activated by NO; this can be considered as a model for oxidized/heme-free sGC. The relaxant effect of BAY 41-2272 and BAY 58-2667 was therefore studied in the gastric fundus and distal colon of apo-sGC mice.

## Methods

Homozygous apo-sGC mice and wild type (WT) controls were derived from a heterozygous breeding (mixed 129/SvJ-C57BL/6J, 11-15 weeks). Circular smooth muscle strips of the gastric fundus and distal colon (after removal of the mucosa) were mounted in Krebs solution (NANC conditions) and incubated with the sGC inhibitor ODQ (10 μM) or its solvent for 30 min. Strips were then pre-contracted with PGF_2α_ and the relaxant effect of cumulatively administered BAY 41-2272 (0.01-10 µM) and BAY 58-2667 (1-100 nM) was examined.

## Results

BAY 41-2272 (from 1 µM on) and BAY 58-2667 (from 1 nM on in the fundus and from 10 nM on in the colon) induced a sustained relaxing response. In WT fundus strips, ODQ reduced the relaxant effect of BAY 41-2272. The relaxing effect of BAY 41-2272 was decreased in fundus strips of apo-sGC mice, but it was not influenced by ODQ. ODQ increased the relaxant effect of BAY 58-2667 in WT fundus strips. The relaxant effect of BAY 58-2667 was more pronounced in fundus strips of apo-sGC mice, but it was not influenced by ODQ. The relaxing effect of BAY 41-2272 and BAY 58-2667 did not significantly differ between colon strips of WT and apo-sGC mice. In both WT and apo-sGC colon strips, ODQ had no influence on the relaxing effect of BAY 41-2272 and BAY 58-2667.

## Conclusion

At the level of the gastric fundus, BAY 58-2667 is more efficient when sGC is in the heme-free condition. At the level of the distal colon, the relaxing effect of BAY 41-2272 and BAY 58-2667 might to a large extent be independent of sGC stimulation/activation.

